# Protective factors for the risk of suffering intimate partner violence in help-seeking women survivors from a social organization in Chile

**DOI:** 10.3389/fsoc.2024.1419182

**Published:** 2024-06-18

**Authors:** Isabel Benjumeda Wynhoven, Carmen Yago Alonso

**Affiliations:** ^1^Facultad de Artes Liberales, Universidad Adolfo Ibáñez, Campus Viña del Mar, Viña del Mar, Chile; ^2^Departamento de Psiquiatría y Psicología Social, Universidad de Murcia, Murcia, Spain

**Keywords:** intimate partner violence, protective factors, Latin America, Chile, women’s health, gender-based violence, prevention factors, risk factors

## Abstract

**Objective:**

Intimate partner violence (IPV) is a major public health problem in Latin America. The present study investigates the protective factors that contribute to minimizing the risk of exposure to IPV analyzing different variables in a sample of Chilean women victims of IPV.

**Methods:**

We used data from the Cicatrices Foundation, a nonprofit Chilean organization providing psychological support to IPV victims. Relevant variables for IPV prevention were identified analyzing a database containing all the information reported by victims during a structured interview. A final sample of 444 women suffering IPV was used in the present study.

**Results:**

Logistic regression analysis was calculated in order to make predictions related to IPV protective factors, showing that having a support network (OR = 2.85), treatment compliance (OR = 2.05) and being younger (OR = 0.95) increased the probability of not living with the aggressor. Another logistic regression analysis was calculated in order to predict IPV victims´ health taking medication intake as an indicator. A significant association was observed between this variable and working outside (*p* = 0.002) and between mediation intake and age (*p* < 0.001), with an OR of 1.987 and 0.93, respectively. Working outside and being younger were identified as protective factors against consuming medication.

**Conclusion:**

To the best of our knowledge, this is one of the first studies conducted in Chile on the prevention of IPV in a sample of victims seeking for help. Our results will contribute to guide policy makers, researchers and other women in the prevention of potential risks for IPV.

## Introduction

Intimate partner violence (IPV) is one of the most frequent forms of violence against women and includes physical, sexual or emotional abuse by an intimate partner (World Health Organization, 2013). Women are vulnerable to suffer it at all stages of their life (Fawole, 2022), regardless of her level of education, nationality, income, religion, age or ethnicity (De Souza et al., 2022). However, the socio-cultural context affects the magnitude and consequences of IPV, with developing countries being more tolerant towards IPV (Zugman et al., 2023) and presenting a lower rate of denunciation (Matheus et al., 2016; Fernández Alonso et al., 2024). Latin America is one of the world ‘s most dangerous regions for women due to its huge rates of IPV (World Health Organization, 2021), which was sharpened by the pandemics (Mejia et al., 2021; De Souza et al., 2022). Chile presented the worst self-perceived health observed in women (López-Contreras et al., 2023) and a significant increase in IPV (Clark et al., 2022).

A factor increasing IPV exposure is living with the aggressor, as shown by statistical femicide data in Chile and Spain (Cantor et al., 2022; Government Office against Gender-based Violence, 2023). Among the factors that contribute to maintaining cohabiting, social support has been extensively studied (MatudAznar et al., 2003; Rivas et al., 2018; Soria, 2020). Isolation and the threat context contributed to avoid report (Government Office against Gender-based Violence, 2015). Decision to abandon the legal procedure correlates with less social support (Abarca, 2013). Being ashamed of failure, developing tolerance towards violent behavior and psychological or economic dependence can contribute to this risk (Eze-Ajoku et al., 2022; Johnson et al., 2022; Mañas et al., 2023). Perceived support is strongly associated with better mental health (Matud et al., 2009; Ogbe et al., 2020).

Education and employment are empowering tools for women, but they do not consistently serve as a protective factor against IPV (Ghoshal et al., 2023). Regarding age, young women are exposed to more partners in a shorter time, being more vulnerable to IPV (Government office against gender-based violence, 2020). However, older women could have a minor risk of suffering IPV because of having more influence and power within the family (Castro et al., 2017; Warwick-Booth and Coan, 2022).

On the other hand, women suffering IPV have feelings of ambivalence and insecurity that make them unable to finish the relationship (Holt et al., 2008). They use more health services and have a worse perception of their wellness (Signorelli et al., 2023). IPV victims present post traumatic symptomatology and emotional dysregulation (Muñoz-Rivas et al., 2021). There is a correlation between substance consumption and IPV. This association may result as a form to confront violence and can in turn increase women’s vulnerability to suffer it (Torres-Lorenzo et al., 2022).

Only a reduced percentage of women do search for help (Labrador et al., 2010) or report violence, with a reduced sample of victims searching for professional help (Alonso et al., 2024). Many women accept the reality of violence they are living (Flury and Nyberg, 2010). Therefore, it is crucial to know which variables are relevant to prevent IPV exposure in highly vulnerable social contexts. Specifically, in Latin-American context, the prevalence of poverty has dramatic consequences in the physical and mental health of women and has a pronounced impact on IPV (Kelly, 2010; Kohrt et al., 2015; Matheson et al., 2015; Romero Bello and González Blanco, 2017; Daugherty et al., 2022). Therefore, IPV is an important health problem with a strong social dimension (Malik et al., 2021). For this, a social and community approach is required, combining both public health and community efforts for a proper response (Alonso and Rodríguez, 2024).

The main objective of the present study was to identify protective factors against IPV in help-seeking women. To do so, variables such as living with the aggressor and medication intake were used in order to make predictions related to IPV protective factors.

Our results will substantially contribute to the creation of a more comprehensive profile of abused women in Chile. All this will provide substantial information to policy makers, researchers, and women in general to prevent them from potential risks for abuse.

## Method

### Study design and data source

A cross-sectional analysis of data was conducted from the data obtained by the Cicatrices foundation in Chile. This organization exists since March 2021 and is devoted at helping IPV victims, offering psychological support, as well as legal and social orientation. The foundation offers free help and operates online and so it reaches the whole country. Participant selection was obtained from the database of women survivors of IPV who voluntarily sought help at the foundation between 2021 and 2023. Out of a total of 1,279 users, those cases lacking information or presenting inconsistent data were excluded. The final analyzed sample consisted of 444 Chilean women suffering IPV. Inclusion criteria considered data from women survivors of IPV whose cases were closed by the time of analysis. Exclusion criteria included women who were in the middle of the intervention process with an open case.

### Procedure

The researchers analyzed an existing data base containing information on IPV survivors from September 2021 to March 2023. Data was collected using a structured instrument based on a Foundation form that was applied by a psychologist and/or social worker from the Institution and contained questions labeled as dichotomic variables (yes/no): whether the survivor took any medication, if she was living with the aggressor, if she was working outside the house, if she had network support and if she finishing the 4 session treatment. Other variables were the survivor’s age and the number of children (quantitative). Finally, educational level was measured as a categorical value (0 no education, 1 basic education, 2 middle education, 3 technical education and 4 university).

After the first interview, one of the psychologists working at the Foundation contacted the survivor and agreed on the session’s dates and the type of online contact (zoom, meet, video call or normal call). The process was backed up in the Sirus database with a summary of each psychological session and the dates. The foundation offers a total of six free sessions. In those cases, in which the user abandoned the treatment, the reasons were specified, the date of closing was stored, and the record was closed. All the information was stored anonymously and treated confidentially.

### Data analysis

Data was analyzed with the Jamovi 2.3.21. Descriptive analysis was conducted. Contingency tables were calculated to know variable associations using Chi square test. A significant contrast value was considered if *p* < 0.05. Logistic regression analyses were applied to identify IPV predictor variables. Probability was measured through the odd ratio analysis (OR), with confidence intervals of 95% (IC).

### Ethical consideration

Before data collection, ethical clearance was obtained from Adolfo Ibáñez University, Chile. The study participants received information on the confidentiality of the study and their rights during data collection. Written informed consent was taken from the participants before data collection was started. Furthermore, the authors were given approval from the Foundation to use the data.

## Results

Table 1 shows the result of selected socio-demographic characteristics of the participants. Approximately 70% of the sample resides in large urban areas, specifically from the metropolitan region of Santiago and Valparaiso. Mean age was 36.6 years and 86.9% had children, with mean 1.87. The prevailing educational level was secondary education (41.1%), followed by university (27.3%) and technical studies (26.3%). At the Foundation, 39.8% required legal derivation which refers to legal procedures related to violence complaints, separation or divorce from the aggressor or alimony. This guidance was provided by a lawyer. On the other hand, 13.5% needed social orientation, referring to any type of guidance related to house rentals, rental aids, or subsidies from the state. This help was provided by a trained social worker.

**Table 1:** Percentage distribution of socio-demographic characteristics of the participants.

Characteristics		%
Place of residence		
	Metropolitan region of Santiago	38.9
	Valparaiso	31.2
	Others (Biobio, Maule, Antofagasta, Libertador B. O’Higgins, De Los Lagos, Coquimbo, etc.)	29.9
Age group		
	Below 30	25.45
	30–39	39.41
	40–49	25.23
	50–59	7.66
	60- older	2.25
Level of education		
	None	0,3
	Basic	5
	Middle	41.1
	Technical	26.3
	University	27.3
Number of children		
	0	13.03
	1	27.87
	2	33.93
	3 or more	25.17
Requested derivation	Legal	39.8
	Social	13.5

In order to identify protective factors that contribute to minimizing the risk of exposure to IPV, different variables such as network support, educational level, working outside, number of children, age, medication intake and finishing the treatment were explored. Living with the aggressor variable was considered as an indicator of IPV exposition.

Support network is present in 72.4% of the sample, mainly concentrated in the family (72.6%). Having a support network and living with the aggressor are not independent variables, showing a statistically significant relationship (χ^2^_1_ = 20.1; *p* < 0.001). Among those women presenting a support network, about 85.7% did not live with the aggressor, whereas 14.3% reported to live with him. The OR indicates that the probability of not living with the aggressor in those women having a support network was 3.11 times higher as compared to those women lacking a support network. So, having a support network appears to be a protective factor for living with the aggressor.

Another variable that was explored in relation to living with the aggressor was treatment compliance. It was observed that the number of women fulfilling the treatment lived at a lower rate with the aggressor (82.9% vs. 17.1%). The result was significant (χ^2^_1_ = 3.73; *p* < 0.05), with an OR of 1.77. Therefore, finishing the treatment increases the probability of not living with the aggressor.

Age was also a significant variable (χ^2^_1_ = 4.66; *p* < 0.003), with an OR of 1.03. So, each year, the probability of living with the aggressor increases by 1.03 times. The number of children was also significant (χ^2^_1_ = 3.60; *p* = 0.05), with a probability of coexistence with the aggressor 1.18 times as the number of children increases. The educational level variable was not a predictor of living with the aggressor (χ^2^_1_ = 0.85; *p* = 0.35).

The four significant variables obtained were used as predictors of living with the aggressor (having support network, treatment compliance, age and number of children). The rest of the variables were discarded for showing no association with the dependent variable. A logistic regression analysis was conducted to build a model that, despite having a low predictive quality (R2McF = 0.09), may offer useful knowledge in maintaining the coexistence with the aggressor. Having a support network, treatment compliance and being younger increased the probability of not living with the aggressor (Table 2). No interaction was observed between the predictor variables.

**Table 2:** Predictors of not living with the aggressor.

						IC 95%	
Predictor	Estimator	EE	*Z*	*p*	Odds ratio	Inferior	Superior
Constant	2.154	0.705	3.05	0.002	8.620	2.164	34.334
Age	−0.051	0.018	−2.87	0.004	0.951	0.919	0.984
With network support	1.046	0.334	3.14	0.002	2.846	1.480	5.471
Compliance treatment	0.719	0.333	2.16	0.031	2.051	1.067	3.943

Reference level for the variables has network support and compliance treatment is No.

Out of the total sample, 44.8% of the users reported taking medication, mainly sleep inducers (zopiclone), antidepressants (sertraline and fluoxetine) and anxiolytics (clonazepam). An elevated percentage of women taking medication were not living with the aggressor (81%), but the relation between medication intake and not living with the aggressor was not significant (*p* = 0.77). In summary, women suffering IPV consume medication even if they are not living with the aggressor anymore.

A significant association obtained was that between medication intake and working outside (*p* = 0.002). The results showed that 62.5% of the women working outside did not take any medication. A logistic regression analysis was performed taking as a predictor working outside and age and as a dependent variable medication intake. The OR was 1.987 between the dependent variable medication intake and the independent variable working outside. This indicates that women working outside were less likely to take medication as compared to those women not working outside. In terms of age, the OR was 0.938 and an inverse relationship was observed. So, older women presented a higher probability of consuming medication, with lower intake observed in younger women (6.2% for each year the user is younger). No interaction was observed between the predictor variables (Table 3).

**Table 3:** Predictors of not taking medication.

						IC 95%	
Predictor	Estimator	EE	*Z*	*p*	Odds ratio	Inferior	Superior
Constant	2.176	0.493	4.41	< 0.001	8.807	3.350	23.153
Age	−0.064	0.013	−4.98	< 0.001	0.938	0.915	0.962
Works outside	0.686	0.229	2.99	0.003	1.987	1.267	3.114

Reference level for the variable works outside is No.

## Discussion

The present article analyzes the profile of 444 women receiving online psychological, social, or legal support due to experiencing IPV. It is remarkable that an important number of users between 18 and 36 years old ask for help (50%). This shows that young women are searching for help, as previously described (Labrador et al., 2010; Matheus et al., 2016). IPV is common in relationships between young people (Sardinha et al., 2022). Age is a significant variable for predicting both living with the aggressor and medication intake, and so, it should be considered both in the early detection and in the professional intervention in order to avoid IPV aggravation.

Among the main findings of our research, having a support network is a protective factor against living with the aggressor. Family appears as a protective factor for women suffering IPV, and may help leaving IPV (Klein and Milardo, 2000; Wright, 2015; Pir et al., 2023). However, social support deserves a deeper exploration, since previous studies informed that isolation might not be a descriptive feature of the victims in all cases (Labrador et al., 2010). On the other hand, social support was not always a protective factor for women. In a study conducted in Nicaragua, it was observed that normalization in the victim’s environment may be an obstacle to escape IPV depending on the socio-cultural context (Rivas et al., 2018).

Consistent with the previous result, we found that living with the aggressor was related to not finishing the psychological treatment. The main reasons for stopping the treatment were not answering the phone or not attending the sessions. Previous research showed that women are normally in charge of house and children (especially in Latin America), as well as working outside, and so they postpone their self-care prioritizing the family (Flake and Forste, 2006). Also, in these countries women are less empowered about their rights (Restrepo et al., 2022).

It is worth mentioning that separation of the aggressor does not imply the end of violence or suffering (Labrador et al., 2010; Karakurt et al., 2014). IPV victims experience burn out, mental health disturbances and often need medication intake (Chandan et al., 2020). In our sample, 44,8% of the women reported to take antidepressants, anxiolytics, or sleep inducers, consistent with previous literature (Clemente-Teixeira et al., 2022). When analyzing the correlation between living with the aggressor and taking medication, we found that 81% of women took medication despite not living with the aggressor. This is consistent with IPV consequences (Malik et al., 2021; Newnham et al., 2022). In the present sample, being younger results in a protective factor against medication intake. When analyzing the association between medication intake and age, older women with an active drug use presented a slightly higher probability of consuming medication as they got older. For each year women suffering IPV get older, the probability of not taking medication decreased 6.2%. However, this association needs to be explored with more detail and a bigger sample that may help to obtain a more robust conclusion.

Together with this, women not working outside consumed more medication as compared to those working outside. So, working outside might be a protective factor against consuming medication but not for living with the aggressor.

Education level, on the other hand, was not significantly associated with living with the aggressor and so it is not necessarily a protective factor against IPV. Previous research showed that IPV rates sharply decrease when women have higher education levels or are at the highest wealth status (Castro et al., 2017). However, as stated before, the socio-cultural context strongly influences these variables, as it occurred with family network support. Neither education nor employment of women are protective factors in themselves (Ghoshal et al., 2023). In patriarchal societies, women having more education than their partners have a higher risk of abuse, because gender roles entail that husbands should have more education than their wives (Flake and Forste, 2006).

According to our results, among the crucial factors that make women search for help and finish treatment were working outside, not living with the aggressor and having social support. Finally, despite offering free psychological attention in our foundation, as well as legal and social orientation, treatment abandonment rate was considerable, which positions this fact as a crucial variable that needs to be studied in more detail in the near future.

## Conclusion

To date, studies related to IPV survivors in Chile are scarce. This research contributes to identifying protective factors for IPV and will contribute to the creation of a more comprehensive profile of abused women. Despite the number of women suffering IPV in Chile is far higher than those searching for help at one Foundation, the analyzed data is a significant sample of the actual situation.

It is important that community intervention programs in IPV are designed following a local perspective and based on data that provides verified information. Variables, such as social support, are key to the IPV prevention, playing a relevant role in one of the highest risk scenarios for women, which is living with the aggressor. However, it is necessary to define what type of support is necessary. As reported from the latest Macrosurvey in Spain (Government office against gender-based violence, 2020), contact with a professional support network is fundamental. Psychological support is crucial to overcome the consequences of violence. However, it is common for women to encounter difficulties that prevent them from accessing or continuing in intervention. Considering the cyclical and intermittent dynamics of the recovery processes makes it necessary to identify the stage in which the woman is in her motivation for change, as described by Prochaska and DiClemente’s Transtheoretical Model of Change in its application to IPV (Manjón & De La Viuda, 2017). It is especially useful to adapt the intervention to the phase of the process that the woman is in to optimize the intervention and prevent abandonment. Risk factors for IPV dramatically increased due to the pandemics and its consequences (unemployment, substance abuse, stress etc.), leading to a collapse of the mental health system in Chile (Bravo, 2022). So, prevention is a dynamic and community work and intervention should focus on psychological, social, educational and health contexts.

As a line of future research, the authors propose to further evaluate protective variables for IPV through the identification of the highest risk contexts.

### Study limitations

This study has a few limitations. First, further research should delve into the type of violence experienced by the survivors to correlate this information with medication intake and with living with the aggressor. The type of social support received is also relevant and should be deeper analyzed.

Also, the results of this study may be affected by the social desirability of the participants, especially in those variables such as medication intake and living with the aggressor. Qualitative data may contribute to solve this, providing more information on the victim’s context.

Finally, it is important to note that the analyzed sample is not representative of all women experiencing IPV, but only of those who have searched for help and are already prepared to break the cycle of violence.

Despite these limitations, this study has contributed significantly to the literature and expands knowledge on the factors that can protect from IPV, being one of the first studies analyzing IPV survivors in the post pandemic context in Chile.

## Data availability statement

The original contributions presented in the study are included in the article/supplementary material, further inquiries can be directed to the corresponding author.

## Ethics statement

The studies involving humans were approved by Universidad Adolfo Ibáñez. Comité Ético de Investigación. The studies were conducted in accordance with the local legislation and institutional requirements. The participants provided their written informed consent to participate in this study.

## Author’s note

The database used in this study belongs to Foundation Cicatrices, to which we are thankful.

## Author contributions

IBW: Data curation, Formal analysis, Investigation, Methodology, Writing – original draft, Writing – review & editing. CYA: Data curation, Formal analysis, Investigation, Methodology, Writing – original draft, Writing – review & editing.
